# Mpox in a Gynecological Setting: A Case Report

**DOI:** 10.7759/cureus.92052

**Published:** 2025-09-11

**Authors:** Yaw Abrefa Safo, Dorothy Sackey

**Affiliations:** 1 Obstetrics and Gynaecology, Tema General Hospital, Tema, GHA; 2 Paediatrics and Child Health, Catholic Hospital Battor, Tema, GHA

**Keywords:** epidermiology, genital rash, ghana, gynecology, monkeypox, mpox, vulval lesions

## Abstract

Monkeypox, now called mpox, is a DNA viral-associated infection with both zoonotic and person-to-person transmission. It typically starts with the onset of prodromal symptoms followed by maculopapular or vesicular rashes at the site of primary infection, which later spreads in a disseminated fashion. The clinical presentation and disease severity of Mpox infection have varied immensely, mostly depending on the viral strain, the route of transmission, host susceptibility, and the quantity of virus inoculated. We present a 24-year-old female patient with no comorbidities who developed multiple vulval rashes and purulent vaginal discharge. Mpox infection from clade II (formerly West African clade) with sub-clade IIa was confirmed by polymerase chain reaction (PCR). The patient was managed with supportive care at our Infectious Disease Centre and later discharged home to continue isolation until all scabs had fallen off. Given the heterogeneous presentation of mpox disease, obstetricians and gynecologists should maintain a high index of suspicion in patients with genital lesions. Establishment and optimization of PCR protocols in the sub-Saharan region are necessary for prompt confirmation and management.

## Introduction

Mpox, formerly called monkeypox, is caused by the mpox virus (MPXV), which is an enveloped virus with a double-stranded DNA genome that belongs to the *Poxviridae *family [1]. It is mostly zoonotic in its spread; however, there is also person-to-person spread. Human disease caused by these clades was first identified in 1970 in the Democratic Republic of the Congo (DRC) [2]. Previously, it was dominant in the eastern corridors of Africa, where these Orthopoxviruses remained zoonotic in wildlife reservoirs with occasional local epidemics [3]. The World Health Organization (WHO) on July 23, 2022, declared mpox as a Public Health Emergency of International Concern (PHEIC) [4]. The majority of mpox cases have mainly been within the corridors of Africa, with the first outbreak outside Africa reported in 2003 in the United States. This first outbreak outside Africa appeared in the United States of America; it was linked to infected rodents that had been imported from Ghana [5]. The African Centre of Disease Control announced in 2024 that about 13 countries within the region had reported 2,863 confirmed cases of mpox with 517 confirmed deaths, and therefore declared a Public Health Emergency of Continental Security. Since 2022, Ghana has continued to have local sporadic epidemics, and as of August 07, 2025, the Ghana Health Service (GHS) had confirmed 328 cases with one death [6]. The first outbreak in Ghana occurred in June 2022 with five cases and has since increased [6]. Whereas this and other mpox cases reported globally have both sexual and non-sexual transmissions, it rarely presents with gynecological symptoms. Mpox infection is confirmed via real-time polymerase chain reaction (PCR) testing of swabs from primary or secondary sites. Other laboratory tests will help differentiate other possible causes. In this case report, we describe a 24-year-old female patient with confirmed mpox who presented with painful, pustular vulval rashes. The clinical manifestation of the disease was typical of mpox. We would therefore like to underscore the importance of clerking and a high index of suspicion in light of this emerging disease by clinicians and, more importantly, obstetricians and gynecologists.

## Case presentation

A 24-year-old female patient reported to the gynecological emergency unit (GEU) of the Tema General Hospital (TGH), Ghana, on 5^th^ July, 2025, with painful itchy vulval rashes of two days’ duration. This was associated with swelling of the vulva and a creamy, copious vaginal discharge, which was non-offensive. She had no other lesions aside from those noted in the genital region. She had no history of close contact with anyone having a similar rash on the body, nor of close contact with a confirmed or suspected case of mpox recently. She had not interacted with any stray animal or pet but had an unprotected sexual encounter with an old male friend 10 days prior to presentation. She could not tell if the male partner was a known HIV patient or not.

Two days post sexual encounter, she recalled having intermittent fever with chills and joint pains, which resolved after intake of non-steroidal anti-inflammatory drugs (NSAIDs). Two days prior to the presentation, she noticed the development of pustular rashes on the vulva, which were pruritic in nature and prominent on both labia majora and minora. It was associated with severe vulvodynia, throbbing in nature, occasionally piercing and radiating deep into the vagina. She rated it 9/10 on the pain score scale, worsened on movement, and interfered with daily activity. This was only relieved by NSAIDs and the application of a soaked, warm towel on the vulva. Twelve to 24 hours later, she noticed a creamy discharge, which became copious over the period prior to presentation. She was para 0; her last normal menstrual cycle was on 24th June, 2025, and lasted for four days with the use of two pads per day. She had no history of recent weight loss, dysmenorrhea, intermenstrual bleeding, post-coital bleeding, or superficial or deep dyspareunia. Her menarche was at 11 years of age, and coitarche was at 20 years of age. She had no known history of pelvic inflammatory disease (PID) and had not had a pap smear done before. She had no known comorbidity, no drug or food allergies, and no vaccination history against varicella and measles viruses. There was no significant family history. She was a non-smoker, occasionally took alcohol, and denied illicit drug use.

On physical examination, the patient was conscious, alert, ambulatory, and had stable vital signs. She was not pale, not jaundiced, and afebrile with a temperature of 36.6°C. She had no generalized lymphadenopathy except for bilateral inguinal lymph nodes, which ranged between 0.5 and 1 cm, were mobile, were not matted together, and were mildly tender.

**Figure 1:**
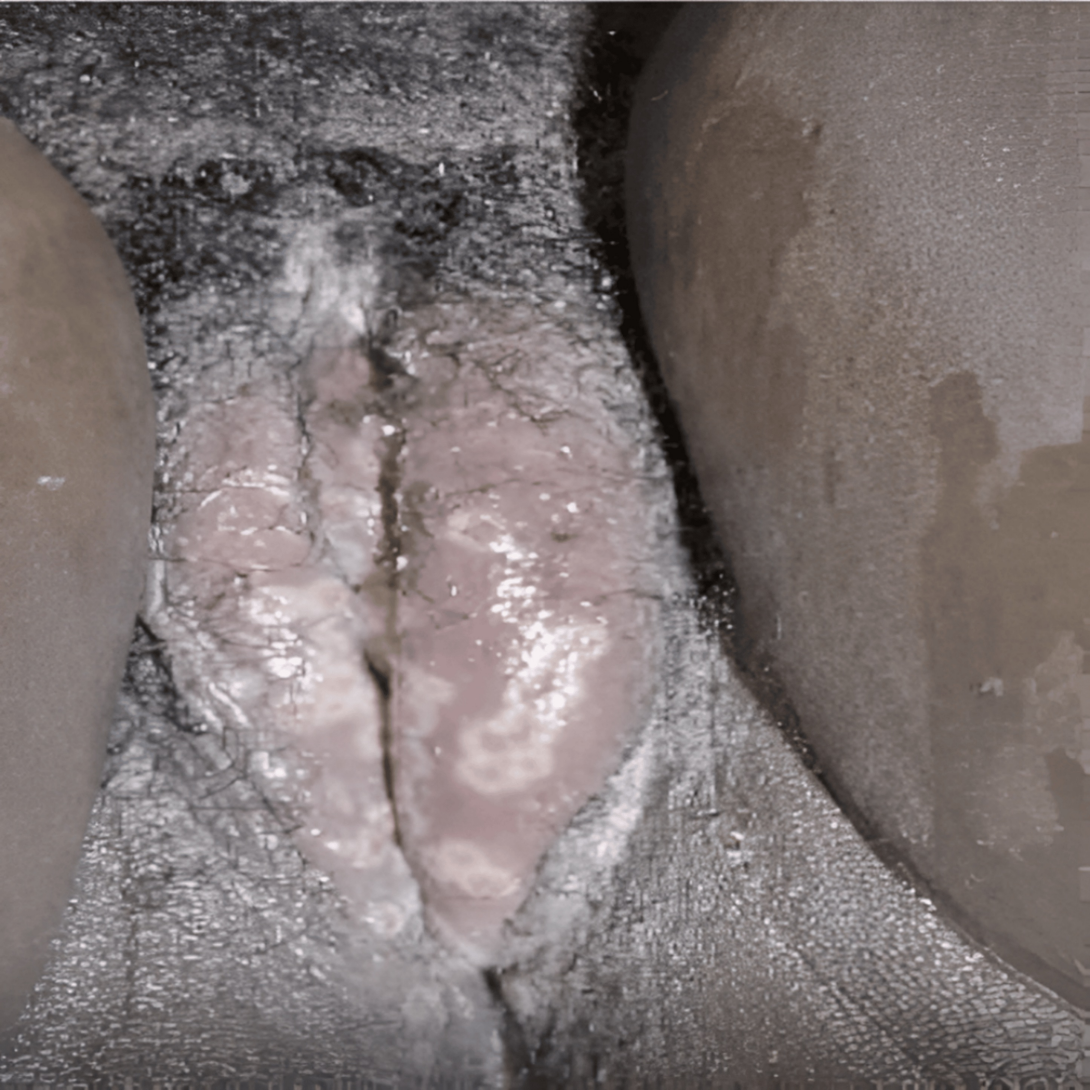
Pustular lesions on both labia majora and minora and the clitoris

**Figure 2:**
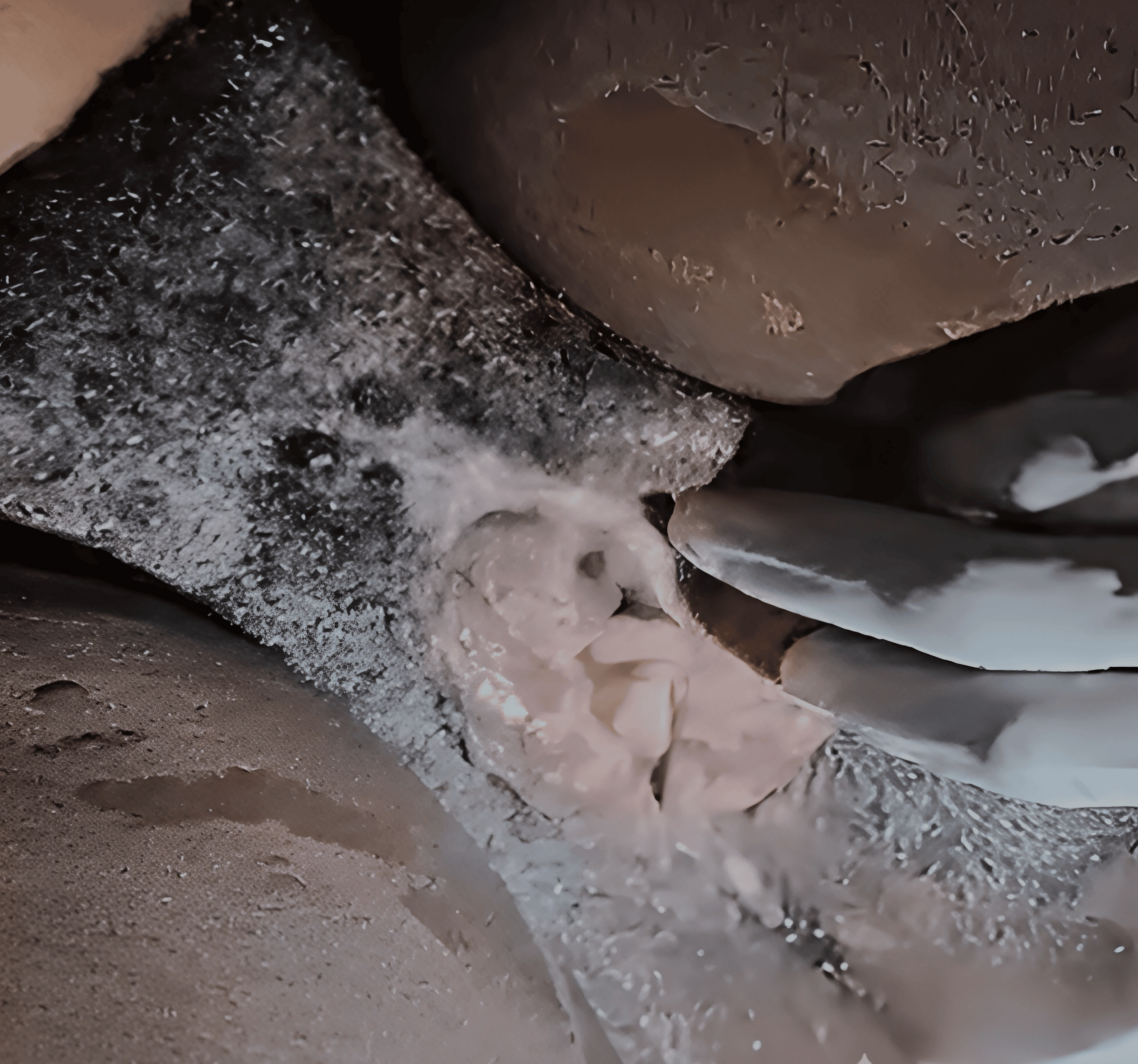
Edematous vulva with excoriations

Status localis examination showed an edematous vulva with bilateral hyperemic labia majora and minora, which were tender on palpation (Figure 1). There were multiple ruptured pustular rashes interspersed with maculopapular forms of about 0.5 cm x 0.5 cm with raised edges (Figure 2). Noted on the vulval skin were excoriations and a creamy, non-offensive discharge. She had a smooth but hyperemic vaginal wall and cervix with no mass felt. No adnexal mass was felt, but she had mild adnexal tenderness and cervical motion tenderness. 

The speculum showed creamy discharge on the vaginal wall and a healthy-looking cervix. A pap smear, high vaginal swab, and endocervical swab were taken for microscopy, culture, and sensitivity. Samples for herpes simplex, retroviral screening, hepatitis B, syphilis screening, baseline full blood count, and kidney and liver function test (LFT) were carried out (Table 1). Diagnoses of PID and suspected vulvar lymphogranuloma venereum (LGV) were made. The following differential diagnoses were considered: HIV, vulval molluscum contagiosum, herpes genitalis, lichen sclerosus genitalis, vulval eczema, vulval psoriasis, vulval intraepithelial neoplasia (VIN), and vulval cancer. She was rehydrated, started on broad-spectrum antibiotics (ceftriaxone, metronidazole, and doxycycline), and admitted. Over the course of 24 hours, the pain was recurrent and intense, requiring an upgrade of her treatment using the WHO Pain Ladder from intravenous paracetamol to intramuscular morphine. Initial laboratory tests showed a normal white cell count of 7.8 x 109/L, with 67% neutrophilia and 31% lymphocytes.

**Table 1:** Laboratory results

TEST	RESULTS
Rapid Plasmin Reagin (RPR)	Negative
Hepatitis B Surface Antigen	Negative
Herpes IgM/IgG	Negative
Retroscreening (HIV 1 and 2)	Negative
*Chlamydia *Antibodies	Negative
Gonococcal Antibodies	Negative
Swab Culture Results	Negative
Blood Urea Estimation (BUE) and Creatinine (Cr)	Urea: 3.1mmol/L, Cr: 66umol/L
Liver Function Test	Aspartate Aminotransferase (AST): 33 U/L; Alanine Aminotransferase (ALT): 39 U/L; Alkaline Phosphatase (ALP): 78 U/L; Gamma-Glutamyl Transferase (GGT): 59 U/L; Total Protein: 6.6g/dL; Bilirubin: 0.8mg/dL
Mpox Reverse Transcription Polymerase Chain Reaction (RT-PCR)	Positive

The lesions on day 2 had started spreading to the thighs and upper limbs, and a differential diagnosis of mpox infection was considered. Dry swab specimens of two lesions (right hand and left thigh) and two wet lesion specimens from the vulva were taken and sent to the Reference Lab (as authorized by Ghana Health Service) for a confirmatory mpox reverse transcription-PCR (RT-PCR) test. A biopsy of the vulva lesions was also carried out.

**Figure 3:**
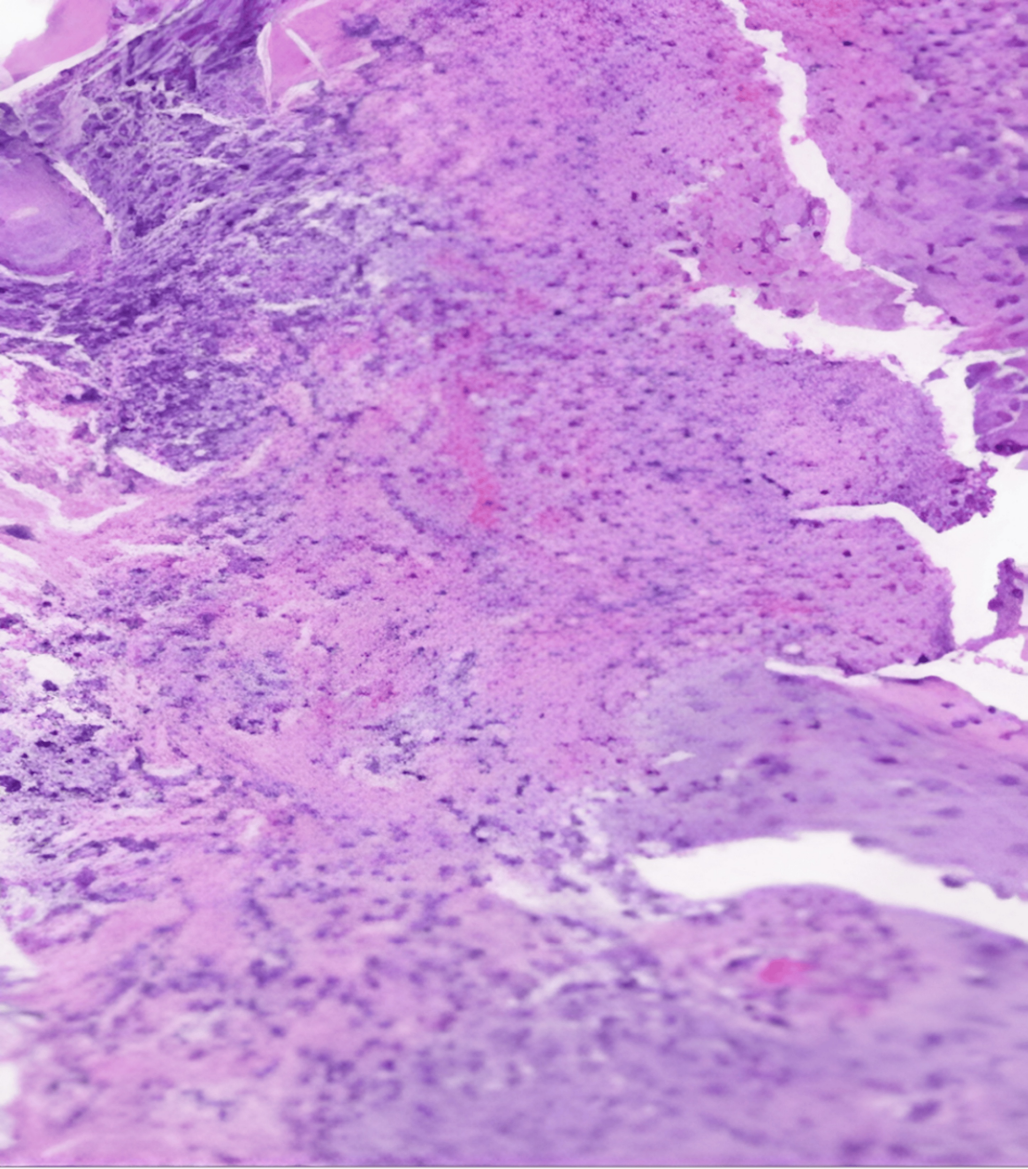
Biopsy results of the mpox lesion with dermal inflammatory cellular layers and multinucleated keratinocytes

All lesions were confirmed to be positive for mpox viral DNA, and she was sent to the Infectious Disease Isolation Centre of the hospital, where she was monitored for two more days before being discharged home for isolation. The biopsy results showed multiple multinucleated keratinocytes and focal to full-thickness epidermal necrosis interspersed with extensive dermal inflammatory cells. Few keratinocytes exhibited cytoplasmic filling with eosinophils, with a “ground glass” appearance consistent with viral infection (Figure 3). 

She was counselled on the condition, its mode of spread, and complications. She was advised to keep the lesions clean and dry using mild soap and moisturizing creams such as shea butter. Again, she was educated to remain in her own room at home, use designated items such as bowls, spoons, towels, etc., without sharing with any member of the household, and avoid contact with immunosuppressed people such as the aged, pregnant women, and infants until the rash has healed completely. It was recommended to her to use face masks at all times and abstain from sexual encounters until the rash has healed completely.

**Figure 4:**
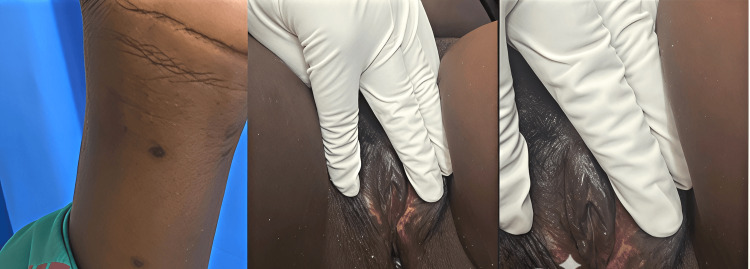
Healed vulva and upper limb lesions

While at home, regular monitoring of the patient’s condition was done via phone until all crusts and scabs had completely disappeared (Figure 4). No complication was reported during the isolation phase, with resolution of all lesions and vaginal discharge. Contact tracing was carried out by the public health personnel of the hospital.

## Discussion

This is a case worthy of note, as it appears to be the first confirmed mpox infection manifesting primarily as a vulval lesion with discharge after mild prodromal symptoms in the sub-region and perhaps beyond. Mpox is rapidly spreading, with the springing up of new cases in the sub-Saharan region, so it is important for all clinicians, including gynecologists, to be abreast with epidemiological and clinical case identification as well as case management of mpox infection. Mpox, a disease caused by an orthopoxvirus, a genus of viruses in the family *Poxviridae *and subfamily *Chordopoxvirinae*, similar to smallpox, is on the rise in Ghana [7].

The ideal mpox case starts with the onset of prodromal symptoms followed by a maculopapular rash from the primary site, which later disseminates to other parts of the body. Typically, it has three stages: an incubation period ranging from seven to 14 days; a prodromal phase characterized by non-specific symptoms of fever, chills, joint pains, headache, and myalgia. The “rash” phase is next, when an initial maculopapular rash progresses to vesicular and later crusted [8]. Even though overall, this case report has followed a similar pattern, her prodromal symptoms were mild, and the main presenting symptom was atypical due to the appearance of vulval lesions alone. The mode of transmission via humans remains as body fluids such as sweat, stool, urine, etc., close contacts, respiratory droplets, and contaminated materials. The atypical presentation of vulva rashes as a standalone or sole primary presentation may be due to a possible high viral load in the vagina, the primary transmission site. A high incidence has been reported among men having sex with men (MSM), people with multiple sexual partners, and people who have sex without condoms, suggesting semen as another medium of body fluid implicated in its transmission. Although the patient had no history of contact with any person with a history of mpox or stray animals, there was a history of sexual encounters prior to the development of prodromal symptoms, indicating close contact during sexual intercourse, and semen via unprotected sex must be related to the spread [8]. 

The vulval pain experienced by the patient was excruciating, similar to what others stated in literature [9]. Consideration for differential diagnoses such as HIV, vulval molluscum contagiosum, LGV, herpes genitalis, lichen sclerosus genitalis, vulval eczema, vulval psoriasis, VIN, and vulval cancer was made, as vulval mpox could mimic these conditions. However, herpes simplex and molluscum contagiosum often present with smaller vesicular lesions, with herpes simplex more limited to dermatomes, while molluscum contagiosum has an indentation at the center of the rash. With respect to syphilis, the nature, distribution, number, and painful state ruled primary syphilis out. Serological testing for diagnosing mpox is limited, as immunologically, there is a cross-reactivity with other orthopoxviruses [10]. However, serology for other diseases such as herpes and LGV is important in ruling out differentials. Histological examination of the biopsied lesion further improved diagnosis, ruling out VIN, vulval cancer, vulval psoriasis, and others.

In Ghana, all suspected and probable cases of mpox are communicated to the Public Health Unit, and samples are collected and sent to the Reference Laboratory for diagnosis by PCR. Once proven, these patients are counselled and isolated at home after treatment of secondary infections, and contact tracing is carried out. In our case, the patient’s hospitalization earlier on was based on allowing room for diagnostic evaluation, adequate pain control, and treatment of secondary infections.

There is currently no treatment for the disease, but adequate counselling, offering supportive treatment, and treatment of superimposed bacterial infection are key to the recovery of the patient. Currently, the CDC has approved Jynneos as the new vaccine against both Mpox and smallpox, which is a two-dose vaccine given four weeks apart [11]. Jynneos is a live viral vaccine created using non-replicating Modified Vaccinia Ankara (MVA) licensed for adults aged ≥ 18 years. The CDC recommends vaccination for people with known or suspected exposure to someone with Mpox, people who had sex with someone in the past two (2) weeks who has been diagnosed with Mpox, MSM, those with occupational exposure to orthopoxviruses, and people travelling to clade 1 outbreak areas with the likelihood of having sex with a partner in these areas.

Outside the scope of vaccination and treatment, public health measures are aimed at minimizing human-to-human transmission via prompt and early recognition of cases. This is based on case definition, a high clinical suspicion, and laboratory tests to confirm suspected cases and containment of the infection through isolation, contact tracing, and public education. In the clinical setting and hospitals, correct infection prevention and control measures to protect hospital staff and other patients are paramount. Such measures should include cleaning and disinfecting all areas used by the infected patient and careful handling of bedsheets, towels, spoons, cups, and other tissues deemed infectious [12].

## Conclusions

Mpox continues to be an emerging problem for Ghana and the sub-region. The rising number of cases and atypical presentations places an onus on physicians, including obstetricians and gynecologists, to remain up-to-date on prompt diagnosis and initiation of appropriate management. Infections via semen and other body fluids, as well as close contact, remain one of the key vehicles for transmission; as such, obstetricians and gynecologists should expect various modes of presentation in their clinics and emergency units. Differential diagnoses of genital rashes, especially pustular and vesicular rashes, should increase suspicion of mpox.

Currently, no available treatment exists, but vaccination of people at high risk of transmission of the virus remains an effective modality of mpox prevention even though it is unavailable in most sub-Saharan African countries. Adequate training on case definition and management should be carried out for all medical professionals, irrespective of specialty.
